# A Study on Clinical Features of Respiratory Syncytial Virus in Acute Respiratory Tract Infection in Elderly Hospitalized Patients in Winter

**DOI:** 10.1155/carj/6631479

**Published:** 2026-02-04

**Authors:** Xuedan Li, Qing Miao, Bingtao Yu, Yanbo Han, Chaofan Feng, Suqin Lu

**Affiliations:** ^1^ Department of Respiratory, Beijing Fengtai YouAnMen Hospital, Beijing, 100069, China; ^2^ Department of Radiology, Beijing Fengtai YouAnMen Hospital, Beijing, 100069, China

**Keywords:** acute respiratory infection, elderly patients, pneumonia, respiratory failure, respiratory syncytial virus

## Abstract

**Background:**

Respiratory syncytial virus (RSV) is one of the important pathogens found in patients with acute respiratory infection (ARI), but there are few studies on RSV infection among elderly people in China.

**Methods:**

This study collected data on the RSV‐infected population among the hospitalized patients with ARI admitted to the Department of Respiratory Medicine of our hospital from November 2023 to February 2024 and analyzed the clinical data of the elderly (≥ 60 years old) grouped by age. Patients admitted to other departments (e.g., general internal medicine, geriatrics) were not included, potentially introducing selection bias toward more severe respiratory presentations.

**Results:**

The total infection rate was 12.16%, and the main symptoms were cough (97.78%), sputum (95.56%), and dyspnea (68.89%); 100% of the patients had comorbidity, and 97.78% of the patients had two or more comorbidities. Laboratory findings were mainly elevated CRP (84.4%), lymphopenia (64.44%), elevated hs‐cTni (51.11%), elevated N‐terminal pro‐B‐type natriuretic peptide (NT‐proBNP) (37.78%), decreased PaO_2_ (88.89%), and elevated PaCO_2_ (44.44%). The rate of mixed bacterial and viral infection was 37.78%; 95.56% of the patients had lung imaging changes; 100% of the patients received respiratory therapy support, and 91.11% of the patients used antibiotics. The discharge rate was 93.33%, and the mortality rate was 6.67%.

**Conclusion:**

There was no obvious abnormal seasonality in the infection rate; the infection rate, the proportion of lymphopenia, and the proportion of respiratory failure were higher than in most other studies. The statistical data are only statistically significant in terms of the decrease in PaO_2_/FiO_2_ (P/F ratio), the elevation in the NT‐proBNP, and some imaging performances. It may be that the number of samples is not large enough to cause the difference to be insignificant. Furthermore, this study exclusively included patients admitted to the Respiratory Medicine Department, which may reflect department‐specific referral bias and potentially underestimate the true burden of RSV in the broader elderly population. Patients admitted to other departments (e.g., general internal medicine, geriatrics) were not included, potentially introducing selection bias toward more severe respiratory presentations.

## 1. Introduction

Respiratory syncytial virus (RSV) is one of the important pathogens identified in patients with acute respiratory infection (ARI) and is increasingly recognized as the cause of infection in high‐risk adults, including those with chronic lung disease and heart disease. Studies on RSV infection have mainly focused on children, and there are few studies on the elderly, especially in China. After the COVID‐19 epidemic, there have been abnormal outbreaks of RSV, such as high morbidity, off‐season outbreaks, multivirus compound infections, and others [1, 2].

This article reviews the data of RSV infection among ARI patients admitted to the Department of Respiratory Medicine of Beijing Fengtai You’anmen Hospital from November 2023 to February 2024 and grouped the elderly (≥ 60 years old) to statistically analyze the infection rate, seasonality, comorbidity, clinical manifestations, laboratory examinations, treatment, and outcome.

## 2. Materials and Methods

### 2.1. Study Population

From November 1, 2023, to February 29, 2024, all patients who were hospitalized in the hospital due to ARI were collected by nasopharyngeal swabs within 24 h of admission and sent for nucleic acid testing of chlamydia, mycoplasma, and respiratory viruses. Patients admitted to other departments (e.g., general internal medicine, geriatrics) were not included, potentially introducing selection bias toward more severe respiratory presentations. RSV patients were screened according to the Diagnosis of Human Respiratory Syncytial Virus Infection (CT/CPMA 028‐2023) [3], and the old patients (≥ 60 years old) were divided into two groups according to age for comparison: the younger‐old group (60–79 years old) and the oldest‐old group (≥ 80 years old). This study was conducted in accordance with the Declaration of Helsinki and was approved by the Medical Ethics Committee of Beijing Fengtai YouAnMen Hospital (20240506), and patient consent statements have been granted an exemption. Written informed consent was obtained from all participants prior to their inclusion in the study. All procedures involving human subjects were performed in compliance with institutional and national ethical standards. No animal experiments were conducted in this study.

### 2.2. Pathogenic Detection

Six types of respiratory virus (RSV, influenza A virus, influenza B virus, adenovirus, parainfluenza virus Type 1, and parainfluenza virus Type 3), were detected by the commercial real‐time fluorescence PCR technology test kit (Respiratory Virus Six‐Fold Test Kit, Beijing Zhuocheng Wison Biological Technology Co., Ltd.). The nucleic acid extraction and detection steps were carried out in strict accordance with the instructions of the kit. The judgment criteria for positive specimens are shown in Table 1.

**TABLE 1:** The judgment criteria for positive specimens.

Specimen	Value
Leukocytopenia	< 3.5 ∗ 10^9^/L

Leukocytosis	> 9.5 ∗ 10^9^/L

Elevated absolute neutrophil count	> 6.3 ∗ 10^9^/L

Elevated absolute neutrophil %	> 75%

Lymphopenia	< 1.1 ∗ 10^9^/L

Decreased lymphocyte %	< 20%

Elevated CRP	> 6 mg/L

Elevated PCT	> 0.5 ng/mL

Elevated ALT	> 50 U/L (male)
	> 40 U/L (female)

Elevated AST	> 40 U/L (male)
	> 35 U/L (female)

Elevated NT‐proBNP	> 900 pg/mL (50–75 years)
	> 1800 pg/mL (> 75 years)

Elevated hs‐cTni	> 0.036 ng/mL (male)
	> 0.02 ng/mL (female)

Abnormal blood gas pH	> 7.45 or < 7.35

Decreased PaO_2_	< 100 − (age ∗ 0.33) ± 5 mmHg

Elevated PaCO_2_	> 45 mmHg

Respiratory failure	PaO_2_/FiO_2_ < 300 mmHg

Mild respiratory failure	200 mmHg < PaO_2_/FiO_2_ < 300 mmHg

Moderate respiratory failure	< 100 mmHg PaO_2_/FiO_2_ < 200 mmHg

Severe respiratory failure	PaO_2_/FiO_2_ < 100 mmHg

### 2.3. Specimen and Data Collection

The following data were collected from the enrolled population: demographic data; comorbidity, including hypertension, stroke, coronary heart disease, heart failure, myocardial infarction, atrial fibrillation, chronic obstructive pulmonary disease (COPD), bronchiectasis, asthma, tumor, renal insufficiency; clinical manifestations, including cough, sputum production, dyspnea, fever, sore throat, fatigue, rhinorrhea, nasal congestion; laboratory examinations on admission, including blood routine, inflammatory markers, liver function, N‐terminal pro‐B‐type natriuretic peptide (NT‐proBNP), hs‐cTni, blood gas analysis; etiological examination; chest CT images before or within 24 h of admission; treatment and outcome.

### 2.4. Statistical Analysis

A continuous variable is expressed as mean ± standard deviation ($\left.\overset{¯}{x}\pm s\right)$, categorical variable is expressed as number and percentage, and inter‐group comparison is carried out by the *χ* ^2^ test, Fisher’s exact test, or Mann–Whitney test. A two‐sided *α* of less than 0.05 was considered statistically significant. Cases with missing data of a variable were excluded from the statistical analyses on this variable. All statistical analyses were done using SPSS statistical software Version 26.0 (SPSS Inc., Chicago, IL, USA).

## 3. Results

### 3.1. Demographic Characteristics

Between November 1, 2023, and February 29, 2024, a total of 443 patients with ARI (aged 73 ± 16.1 years, 227 males and 217 females) were hospitalized, including 370 patients aged ≥ 60 years (aged 79 ± 10.0 years, 187 males and 183 females), among whom 45 patients (aged 82 ± 10.8 years, 21 males and 24 females) met the diagnostic criteria for RSV infection and were finally included in this study. The 45 patients were divided into two groups by age: the younger‐old group (60–79 years old, aged 70.2 ± 6.23 years, 9 males, 8 females) and the oldest‐old group (≥ 80 years old, aged 88.7 ± 5.66 years, 12 males, 16 females).

### 3.2. RSV Infection Rate of ARI Hospitalized Patients

The RSV infection rate is calculated on a monthly basis, as shown in Table 2. Specifically, RSV activity began at a low level in November (1.47%), increased in December (7.00%), peaked in January (24.11%), and then declined in February (11.11%), demonstrating a clear winter seasonal pattern in this elderly hospitalized population. The differences in the two groups of people in different months and the total RSV examination rate were not statistically significant.

**TABLE 2:** RSV infection rate *n*/total (%).

	60–79 years.	≥ 80 years.	Total	*p* value
2023.11	0/36 (0.00)	1/32 (3.13)	1/68 (1.47)	0.471
2023.12	5/55 (9.09)	2/45 (4.44)	7/100 (7.00)	0.609
2024.1	7/46 (15.22)	20/66 (30.3)	27/112 (24.11)	0.066
2024.2	5/48 (10.42)	5/42 (11.90)	10/90 (11.11)	1.000
Total	17/185 (9.19)	28/185 (15.14)	45/370 (12.16)	0.080

*Note:* Data are $\overset{¯}{x}\pm s$ or *n* (%). *p* values were calculated by the Mann–Whitney test, *χ* ^2^ test, or Fisher’s exact test, as appropriate.

### 3.3. Comorbidity and Clinical Manifestation

All cases had comorbidity, and all of them were at high risk of RSV infection. Among them, hypertension (66.67%), stroke (57.78%), diabetes (40.00%), coronary heart disease (37.78%), and heart failure (35.56%) accounted for the top five. 44 patients (97.78%) had two or more comorbidities. The main clinical manifestations were cough (97.78%), sputum production (95.56%), dyspnea (68.89%), and fever (57.78%); the difference in clinical manifestations between the two groups was not statistically significant, as shown in Table 3.

**TABLE 3:** Comorbidity and clinical manifestation *n* (%).

	60–79 years (*n* = 17)	≥ 80 years (*n* = 28)	Total (*n* = 45)	*p* value
*Comorbidity*				
Hypertension	12 (70.59)	18 (64.29)	30 (66.67)	0.664
Stroke	6 (35.29)	20 (71.43)	26 (57.78)	0.017
Diabetes	8 (47.06)	10 (35.71)	18 (40.00)	0.451
Coronary heart disease	3 (17.65)	14 (50.00)	17 (37.78)	0.030
Heart failure	5 (29.41)	11 (39.29)	16 (35.56)	0.502
COPD	6 (35.29)	5 (17.86)	11 (24.44)	0.336
Tumor	6 (35.29)	5 (17.86)	11 (24.44)	0.336
Renal insufficiency	2 (11.76)	4 (14.29)	6 (13.33)	1.000
Atrial fibrillation	1 (5.88)	3 (10.71)	4 (8.89)	0.990
Bronchiectasis	1 (5.88)	2 (7.14)	3 (6.67)	1.000
Myocardial infarction	0 (0.00)	0 (0.00)	0 (0.00)	0.001
Asthma	2 (11.8)	3 (10.7)	5 (11.1)	1.000

*Clinical manifestation*				
Cough	16 (94.12)	28 (100.00)	44 (97.78)	0.378
Sputum production	15 (88.24)	28 (100.00)	43 (95.56)	0.137
Dyspnea	12 (70.59)	19 (67.86)	31 (68.89)	0.848
Fever	8 (47.06)	18 (64.29)	26 (57.78)	0.257
Sore throat	4 (23.53)	4 (14.29)	8 (17.78)	0.701
Fatigue	0 (0.00)	4 (14.29)	4 (8.89)	0.275
Rhinorrhea	0 (0.00)	1 (3.57)	1 (2.22)	1.000
Nasal congestion	1 (5.88)	0 (0.00)	1 (2.22)	0.378

*Note:* Data are $\overset{¯}{x}\pm s$ or *n* (%). *p* values were calculated by the Mann–Whitney test, *χ* ^2^ test, or Fisher’s exact test, as appropriate.

### 3.4. Laboratory Examination

There were 38 cases of an increase in CRP (84.4%), 32 cases of a decrease in the lymphocyte percentage (71.11), and 29 cases of a decrease in the absolute value of lymphocytes (64.44%); ALT increased in 3 cases (6.67%), AST increased in 10 cases (22.22%), NT‐proBNP increased in 17 cases (37.78%), and hs‐cTni increased in 23 cases (51.11%); there were 40 cases of a decrease in PaO_2_ (88.89%) and 20 cases of an increase in PaCO_2_ (44.44%). 27 cases had respiratory failure (60.00%). The data of two groups were statistically significant in respiratory failure count, moderate respiratory failure count, NT‐proBNP value, and AST value.

There were 12 cases of mixed infections with 2 pathogens (26.67%) and 5 cases of mixed infections with 3 pathogens (11.11%). The main pathogens were *Klebsiella pneumoniae* (5 cases, 11.1%), influenza A virus (4 cases, 8.89%), and *Haemophilus influenzae* (4 cases, 8.89%).

Forty three cases (95.56%) had lung imaging changes, mainly bronchial wall thickening, ground‐glass opacity and patchy exudate. Among them, 36 cases (80.00%) had bilateral changes and 7 cases (15.56%) had unilateral changes. The unilateral infiltration data of two groups was statistically significant, as shown in Table 4.

**TABLE 4:** Laboratory examination *n* (%).

	60–79 years (*n* = 17)	≥ 80 years (*n* = 28)	Total (*n* = 45)	*p* value
*Blood routine*				
Leukocytosis	2 (11.76)	3 (10.71)	5 (11.11)	0.248
Leukocytopenia	7 (41.18)	6 (21.43)	13 (28.89)	1.000
Abnormal leukocyte	9 (52.94)	9 (32.14)	18 (40.00)	0.566
Elevated absolute neutrophil count	8 (47.06)	11 (39.29)	19 (42.22)	0.594
Elevated absolute neutrophil %	9 (52.94)	17 (60.71)	26 (57.78)	0.553
Lymphopenia	11 (64.71)	18 (64.29)	29 (64.44)	0.452
Decreased lymphocyte %	12 (70.59)	20 (71.43)	32 (71.11)	0.516
Elevated CRP	15 (88.24)	23 (82.14)	38 (84.44)	0.250
Elevated PCT	3 (17.65)	6 (21.43)	9 (20.00)	0.101
Abnormal blood gas pH	7 (41.18)	11 (39.29)	18 (40.00)	0.922
Decreased PaO_2_	14 (82.35)	26 (92.86)	40 (88.89)	0.364
Elevated PaCO_2_	8 (47.06)	12 (42.86)	20 (44.44)	0.200
Severe respiratory failure	1 (5.88)	0 (0.00)	1 (2.22)	0.001
Moderate respiratory failure	3 (17.65)	3 (10.71)	6 (13.33)	0.046
Mild respiratory failure	6 (35.29)	14 (50.00)	20 (44.44)	0.137
Respiratory failure	10 (58.82)	17 (60.71)	27 (60.00)	0.031
NT‐proBNP	4080.3 ± 9756.58	3966.0 ± 7023.8	4011.2 ± 8101.32	0.019
ALT	17.6 ± 9.19	30.0 ± 54.21	25.3 ± 43.26	0.972
AST	20.5 ± 14.66	34.3 ± 37.04	29.1 ± 31.08	0.017
hs‐cTni	0.06 ± 0.105	0.04 ± 0.034	0.05 ± 0.069	0.355

*Mixed bacterial and viral infection (other than RSV)*				
None	12 (70.59)	16 (57.14)	28 (62.22)	0.367
Mixed 1	4 (23.53)	8 (28.57)	12 (26.67)	0.982
Mixed 2	1 (5.88)	4 (14.29)	5 (11.11)	0.704

*Type of mixed infection*				
Bacteria	2 (11.76)	10 (35.71)	12 (26.67)	0.157
Viruses	2 (11.76)	1 (3.57)	3 (6.67)	0.651
Bacteria and viruses	1 (5.88)	1 (3.57)	2 (4.44)	1.000

*Infection pathogens*				
*Klebsiella pneumoniae*	0 (0.00)	5 (17.86)	5 (11.11)	0.174
Influenza A virus	2 (11.76)	2 (7.14)	4 (8.89)	1.000
*Haemophilus influenzae*	0 (0.00)	4 (14.29)	4 (8.89)	0.275
*Streptococcus pneumoniae*	1 (5.88)	2 (7.14)	3 (6.67)	1.000
*Pseudomonas aeruginosa*	2 (11.76)	0 (0.00)	2 (4.44)	0.137
*Escherichia coli*	0 (0.00)	1 (3.57)	1 (2.22)	1.000
*Staphylococcus aureus*	0 (0.00)	1 (3.57)	1 (2.22)	1.000
*Proteus mirabilis*	0 (0.00)	1 (3.57)	1 (2.22)	1.000
Coronavirus	1 (5.88)	0 (0.00)	1 (2.22)	0.378

*Radiographic imaging*				
Bronchial wall thickening	10 (58.82)	20 (71.43)	30 (66.67)	0.384
Ground‐glass opacity	4 (23.53)	9 (32.14)	13 (28.89)	0.780
Patch exudation	10 (58.82)	17 (60.71)	27 (60.00)	0.900
Negative	0 (0.00)	2 (7.14)	2 (4.44)	0.519
Unilateral infiltration	6 (35.29)	1 (3.57)	7 (15.56)	0.015
Bilateral infiltration	11 (64.71)	25 (89.29)	36 (80.00)	0.106

*Note:* Data are $\overset{¯}{x}\pm s$ or *n* (%). *p* values were calculated by the Mann–Whitney test, *χ* ^2^ test, or Fisher’s exact test, as appropriate.

### 3.5. Treatment and Outcome

All cases received respiratory therapy support of different durations and methods, including 34 cases (75.56%) with nasal cannula oxygen, 5 cases (11.11%) with high‐flow humidification therapy device, and 6 cases (13.33%) with mechanical ventilation, among which 3 cases (6.67%) were noninvasive and 3 cases (6.67%) were invasive. Antibiotics were used in 44 cases (97.78%), intravenous corticosteroids were used in 17 cases (37.78%), and nebulized therapy was used in 38 cases (84.44%).

Forty‐two cases (93.33%) were discharged, and 3 cases (6.67%) died. Days of hospitalization were 12.39 ± 8.11 days, as shown in Table 5.

**TABLE 5:** Treatment and outcome *n* (%).

	60–79 years (*n* = 17)	≥ 80 years (*n* = 28)	Total (*n* = 45)	*p* value
*Treatment*				
Nasal cannula oxygen	12 (70.59)	22 (78.57)	34 (75.56)	0.805
High‐flow humidification therapy device	3 (17.65)	2 (7.14)	5 (11.11)	0.550
Noninvasive mechanical ventilation	2 (11.76)	1 (3.57)	3 (6.67)	0.651
Invasive mechanical ventilation	0 (0.00)	3 (10.71)	3 (6.67)	0.435
Antibiotics	17 (100.00)	27 (96.43)	44 (97.78)	1.000
Intravenous corticosteroids	6 (35.29)	11 (39.29)	17 (37.78)	0.789
Nebulized therapy	14 (82.35)	24 (85.71)	38 (84.44)	1.000

*Outcome*				
Discharged	16 (94.12)	26 (92.86)	42 (93.33)	1.000
Died	1 (5.88)	2 (7.14)	3 (6.67)	1.000
Days of hospitalization	11.36 ± 7.38	9.56 ± 5.68	12.39 ± 8.11	

*Note:* Data are $\overset{¯}{x}\pm s$ or *n* (%). *p* values were calculated by the Mann–Whitney test, *χ* ^2^ test, or Fisher’s exact test, as appropriate.

## 4. Discussion

Five findings were reported in this study: (1) The antiseasonal epidemic situation of RSV caused by the epidemic and control mode of COVID‐19 has passed, and the seasonality of prevalence has returned to normal; (2) the RSV infection rate among elderly ARI hospitalized patients is higher than previously reported; (3) the proportion of lymphocyte decline and respiratory failure was higher than that previously reported; (4) the proportion of heart failure and respiratory failure in elderly RSV patients increases with age; and (5) the laboratory examination, imaging examination, and treatment scheme of elderly RSV patients have their obvious characteristics, which should be paid special attention to.

RSV belongs to the Pneumoviridae family and the Orthopneumovirus genus and is one of the most important viral pathogens causing acute lower respiratory tract infections in children under 5 years old, the elderly, and immunocompromised people. A 4‐year prospective cohort study showed that RSV infection is observed in 3%–7% of healthy elderly people and 4%–10% of adults with chronic cardiopulmonary diseases every year [4]. The seasonality of RSV in China is characterized by starting in November, peaking from December to February, and ending in April of the following year [5]. During the COVID‐19 epidemic, nonpharmacological interventions (NPI) disrupted the typical seasonality of infections with other common respiratory pathogens, and outbreaks of RSV were reported in many places after relaxation (usually several months later) [1, 2]. In this study, RSV accounted for the second place of the main six respiratory virus detection rates and peaked in January and February, which is consistent with the seasonal transmission characteristics before the COVID‐19 epidemic, and no out‐of‐season epidemics were found. Considering that the NPI has been relaxed for more than a year, the prevalence of RSV may be close to normal.

The infection rate in the peak month (January) is 24.11%, close to the 26.5% reported by He [6]. The 4‐month average infection rate was 12.16%, which was higher than the corresponding data in Germany (2.9%∼9.1%) [7] and the United Kingdom (9.6%) [8]. This may be due to the fact that PCR laboratories have become more popular in recent years, and more medical institutions are using PCR to replace the previous rapid antigen test technology and improve the infection rate of RSV. RSV infection in adults has been reported to increase with age [9], but this study compared the infection rate of RSV in two age groups, and there was no statistical difference, which may be related to the short duration of the study and the small sample size.

We observed that cough (97.78%), sputum production (95.56%), and dyspnea (68.89%) were the main manifestations of RSV infection in the elderly in the early stages of the disease. The fever rate (57.78%) is low, and the proportion of upper respiratory tract symptoms such as sore throat, rhinorrhea, and nasal congestion is less than 20%, which was consistent with the rapid progression of the disease and the symptoms of wheezing, shortness of breath, and dyspnea reported in the literature [3, 10]. More than half of the elderly RSV‐infected patients in this study finally developed dyspnea during the clinical course, which is consistent with Luo’s report (68.89% vs. 52.6%) [11]. The reason is that the respiratory system is easily involved after viral infection, causing airway obstruction accompanied by infiltration around the bronchi and mucous membrane edema, bronchial smooth muscle spasm, and subsequent high airway reactivity; secondly, a number of studies have found that among elderly RSV‐infected people, comorbidities are more common [12]. All cases in this study had one or more comorbidities (cardiovascular and cerebrovascular diseases, chronic respiratory diseases, diabetes, etc.), and RSV was the main cause of the exacerbation of airway diseases such as asthma and COPD.

Patients with comorbidities like cardiovascular disease and chronic lung disease are more likely to develop symptoms of RSV disease [13]. For elderly patients, the infection rate of RSV in patients with COPD, asthma, diabetes, and heart failure is much higher than those who have no such conditions, which is 3.51∼13.41 times, 2.27∼2.52 times, 2.35∼6.44 times, 3.75∼6.46 times, and 3.99∼7.63 times, respectively [14]. After RSV infection, it will aggravate the original respiratory and cardiovascular diseases, induce respiratory failure, heart failure, and others.

In this study, 37.78% of patients had elevated NT‐proBNP, and 33.33% of patients had elevated hs‐cTni; this may be related to myocardial injury and cardiac insufficiency caused by cardiovascular system involvement after RSV infection. We also performed blood routines, inflammatory indicator tests, liver function tests, blood gas analysis, and other laboratory tests on all patients. The results were: (1) 29 cases (64.44%) had lymphopenia, which was significantly higher than the blood analysis results of RSV‐infected children of all ages reported by Luo et al. [11] and RSV‐infected people of all ages reported by Gong et al. [15], and (2) 27 cases (60.00%) had respiratory failure; this is higher than the infection rate of respiratory failure in elderly RSV patients in Beijing (15.8%) [11] and close to the data reported in Hong Kong (52.8%) [16]. The elderly are immunocompromised, and the condition is more severe after infection, especially for the high‐risk groups with cardiopulmonary and vascular diseases. In addition, RSV infection is usually associated with worsening of heart failure and respiratory failure. Therefore, in the high infection season, RSV should be regarded as a key surveillance project for elderly high‐risk groups.

In this study, 17 cases (47.78%) had mixed pathogen infections at the time of admission, including 14 cases (31.11%) of bacterial infections and 5 cases (11.11%) of viral infections. The proportion of bacterial infections is significantly higher than that of viral infections, and replication in highly differentiated apical ciliated cells in the human airway after RSV infection is considered to be associated [17], resulting primarily in superficial damage to the inner layer of the airways, which predisposes patients to secondary bacterial infections [18]. The low proportion of RSV merging with other viruses may be consistent with the so‐called viral interference phenomenon, in which one virus prevents the growth of the other virus in the case of co‐infection with multiple viruses [19, 20].

Imaging findings of pneumonia are quite variable and usually mildly abnormal, including unilateral or bilateral patchy subsegmental alveolar infiltrates and rarely lobar consolidation. Diffuse interstitial infiltrates, often considered the classic pattern of viral pneumonia, are uncommon (< 1%) [21]. In this study, 2 patients had negative lung imaging, and after CT examination of RSV infection, it showed an airway‐centered disease pattern, accompanied by bronchial wall thickening, ground‐glass opacity, and small focal consolidation. Bilateral lesions were common, and the imaging manifestations were nonspecific.

The treatment of infection was mainly based on supporting symptomatic treatment. All cases received respiratory therapy support for different durations and methods, and the proportion of mechanical ventilation was basically the same as the literature reported (12.5%) [11]. The high antibiotic prescribing rate (97.78%) likely reflects diagnostic uncertainty and concern for bacterial superinfection, particularly in patients with COPD and imaging abnormalities. However, only 31.11% had confirmed bacterial co‐infection, suggesting potential overuse. This highlights the importance of considering more precise diagnostic tools, such as procalcitonin‐guided protocols, to reduce unnecessary antibiotic exposure and enhance antimicrobial stewardship in similar cohorts. Antibiotics were used in 41 cases (91.11%) and intravenous corticosteroids in 17 cases (37.78%), which was higher than reported, especially antibiotic use (91.11% vs. 77%) [22]. The reason is the lack of specificity in the imaging of patients with RSV infection, and the elderly patients with RSV infection often have comorbidities, especially respiratory diseases. Doctors always treat patients empirically with antibiotics after admission, which increases the rate of antibiotic use. Indications for antibiotic use need to be further standardized. In addition, data on vaccination status (influenza, pneumococcal, COVID‐19) and frailty‐related outcomes (e.g., activities of daily living, Barthel Index, presence of dementia) were not collected, which may represent important unmeasured confounders, particularly in the ≥ 80‐year‐old group.

## Author Contributions

Xuedan Li is responsible for the definition of intellectual content, literature research, data analysis, statistical analysis, manuscript preparation and editing; Qing Miao is responsible for the guarantor of integrity of the entire study, study concepts and design, manuscript review; Bingtao Yu is responsible for the data analysis; Yanbo Han is responsible for the clinical studies, experimental studies; and Chaofan Feng and Suqin Lu are responsible for the data acquisition.

## Funding

The authors received no specific funding for this work.

## Disclosure

All authors read and approved the final manuscript.

## Ethics Statement

This study was conducted in accordance with the Declaration of Helsinki and was approved by the Medical Ethics Committee of Beijing Fengtai YouAnMen Hospital (20240506), and patient consent statements have been granted an exemption. Written informed consent was obtained from all participants prior to their inclusion in the study. All procedures involving human subjects were performed in compliance with institutional and national ethical standards. No animal experiments were conducted in this study.

## Consent

Please see the Ethics Statement.

## Conflicts of Interest

The authors declare no conflicts of interest.

## Data Availability

All data generated or analyzed during this study are included in this published article. Further inquiries can be directed to the corresponding author.

## References

[1] Eden J. S. , Sikazwe C. , Xie R. et al., Off-Season RSV Epidemics in Australia After Easing of COVID-19 Restrictions, Nature Communications. (2022) 13, no. 1, 10.1038/s41467-022-30485-3. PMC9130497 35610217

[2] Billard M. N. , van de Ven P. M. , Baraldi B. , Kragten-Tabatabaie L. , Bont L. J. , and Wildenbeest J. G. , International Changes in Respiratory Syncytial Virus (RSV) Epidemiology During the COVID-19 Pandemic: Association With School Closures, Influenza and Other Respiratory Viruses. (2022) 16, no. 5, 926–936, 10.1111/irv.12998. 35733362 PMC9343326

[3] CPM Association , Diagnosis of Human Respiratory Syncytial Virus Infection (CT/CPMA 028-2023), Chinese Journal of Practical Diagnosis and Treatment. (2023) 37, no. 6, 541–547. 10.3760/cma.j.cn112150-20230522-00399 37482732

[4] Falsey A. R. and Walsh E. E. , Respiratory Syncytial Virus Infection in Elderly Adults, Drugs and Aging. (2005) 22, no. 7, 577–587, 10.2165/00002512-200522070-00004, 2-s2.0-24044538547. 16038573 PMC7099998

[5] Obando-Pacheco P. , Justicia-Grande A. J. , Rivero-Calle I. et al., Respiratory Syncytial Virus Seasonality: A Global Overview, The Journal of Infectious Diseases. (2018) 217, no. 9, 1356–1364, 10.1093/infdis/jiy056, 2-s2.0-85050489387. 29390105

[6] He Y. J. , 2018–2019 Analysis of Human Rhinovirus and Respiratory Syncytial Virus and Adenovirus Infections in Jinan, Chinese Journal of Experimental and Clinical Virology. (2023) 37, 30–38.

[7] Ambrosch A. , Doris L. , Frank K. , and Michael K. , Corrigendum to Focusing on Severe Infections With the Respiratory Syncytial Virus (RSV) in Adults: Risk Factors, Symptomatology and Clinical Course Compared to Influenza A/B and the Original SARS-CoV-2 Strain: Journal of Clinical Virology, 161 (2023), 105399, Journal of Clinical Virology. (2023) 164, 10.1016/j.jcv.2023.105443. PMC10098369 37059614

[8] Walsh E. E. , Peterson D. R. , and Falsey A. R. , Is Clinical Recognition of Respiratory Syncytial Virus Infection in Hospitalized Elderly and High-Risk, The Journal of Infectious Diseases. (2007) 195, no. 7, 1046–1051, 10.1086/511986, 2-s2.0-33947386270. 17330796

[9] Jiang M. Y. , Research Progress on Disease Burden Caused by Respiratory Syncytial Virus Infection in Older Adults, Chinese Journal of Preventive Medicine. (2023) 57, no. 1, 63–69. 36655260 10.3760/cma.j.cn112150-20220721-00742

[10] Chinese Children’s Action Initiative for the Prevention and Control of Human Respiratory Syncytial Virus Infection, Chinese Medical Journal. (2021) 101, no. 36, 2861–2866.

[11] Luo M. , Gong C. , Zhang Y. et al., Comparison of Infections With Respiratory Syncytial Virus Between Children and Adults: A Multicenter Surveillance From 2015 to 2019 in Beijing, China, European Journal of Clinical Microbiology & Infectious Diseases. (2022) 41, no. 12, 1387–1397, 10.1007/s10096-022-04492-7. 36197575 PMC9533982

[12] Malosh R. E. , Martin E. T. , Callear A. P. et al., Respiratory Syncytial Virus Hospitalization in Middle-Aged and Older Adults, Journal of Clinical Virology. (2017) 96, 37–43, 10.1016/j.jcv.2017.09.001, 2-s2.0-85029658530. 28942341 PMC5889293

[13] Mehta J. , Walsh E. E. , Mahadevia P. J. , and Falsey A. R. , Risk Factors for Respiratory Syncytial Virus Illness Among Patients With Chronic Obstructive Pulmonary Disease, COPD: Journal of Chronic Obstructive Pulmonary Disease. (2013) 10, no. 3, 293–299, 10.3109/15412555.2012.744741, 2-s2.0-84878521914. 23536980

[14] Branche A. R. , Saiman L. , Walsh E. E. et al., Incidence of Respiratory Syncytial Virus Infection Among Hospitalized Adults, 2017–2020, Clinical Infectious Diseases. (2022) 74, no. 6, 1004–1011, 10.1093/cid/ciab595. 34244735

[15] Gong C. , Wang X. , Luo M. et al., Risk Factor Analysis of Severe Respiratory Syncytial Virus Pneumonia, Disease Surveillance. (2020) 35, no. 7, 613–617.

[16] Lee N. , Chan M. C. , Lui G. C. et al., High Viral Load and Respiratory Failure in Adults Hospitalized for Respiratory Syncytial Virus Infections, The Journal of Infectious Diseases. (2015) 212, no. 8, 1237–1240, 10.1093/infdis/jiv248, 2-s2.0-84943410318. 25904604 PMC7539363

[17] Zhang L. , Peeples M. E. , Boucher R. C. , Collins P. L. , and Pickles R. J. , Respiratory Syncytial Virus Infection of Human Airway Epithelial Cells Is Polarized, Specific to Ciliated Cells, and Without Obvious Cytopathology, Journal of Virology. (2002) 76, no. 11, 5654–5666, 10.1128/jvi.76.11.5654-5666.2002, 2-s2.0-0036094947. 11991994 PMC137037

[18] Damasio G. A. , Pereira L. A. , Moreira S. D. , Duarte dos Santos C. N. , Dalla-Costa L. M. , and Raboni S. M. , Does Virus-Bacteria Coinfection Increase the Clinical Severity of Acute Respiratory Infection?, Journal of Medical Virology. (2015) 87, no. 9, 1456–1461, 10.1002/jmv.24210, 2-s2.0-84937073114. 25976175 PMC7166438

[19] Liu Y. N. , Zhang Y. F. , Xu Q. et al., Chinese Center for Disease Control and Prevention Etiology Surveillance Study Team of Acute Respiratory Infections. Infection and Co-Infection Patterns of Community-Acquired Pneumonia in Patients of Different Ages in China From 2009 to 2020: A National Surveillance Study, The Lancet Microbe. (2023) 4, no. 5, e330–e339, 10.1016/S2666-5247(23)00031-9. 37001538 PMC12514336

[20] Piret J. and Boivin G. , Viral Interference Between Respiratory Viruses, Emerging Infectious Diseases. (2022) 28, no. 2, 273–281, 10.3201/eid2802.211727. 35075991 PMC8798701

[21] Haber N. , Respiratory Syncytial Virus Infection in Elderly Adults, Medecine et Maladies Infectieuses. (2018) 48, no. 6, 377–382, 10.1016/j.medmal.2018.01.008, 2-s2.0-85043463111. 29548714

[22] Nam H. H. and Ison M. G. , Respiratory Syncytial Virus Infection in Adults, BMJ. (2019) 366, 10.1136/bmj.l5021, 2-s2.0-85072015699. 31506273

